# Evaluation of Fibroblast Growth Factor Receptor 3 (FGFR3) and Tumor Protein P53 (TP53) as Independent Prognostic Biomarkers in High-Grade Non-muscle Invasive Bladder Cancer

**DOI:** 10.7759/cureus.65816

**Published:** 2024-07-31

**Authors:** Anil Kumar, Vivek K Singh, Vishwajeet Singh, Mukul K Singh, Ashutosh Shrivastava, Dinesh K Sahu

**Affiliations:** 1 Urology, King George's Medical University, Lucknow, IND; 2 Center for Advance Research, Faculty of Medicine, King George's Medical University, Lucknow, IND; 3 Central Research Facility/Molecular Biology, Post Graduate Institute of Child Health, Noida, IND

**Keywords:** low grade, high grade, bladder cancer, recurrence, methylation

## Abstract

Introduction

Bladder cancer is a significant health issue with an increased recurrence and progression rate, requiring invasive follow-up, which shows a poor prognosis. In addition, the prognostic role of mutant fibroblast growth factor receptor 3 (FGFR3) and tumor protein P53 (TP53) is controversial; therefore, we investigated the methylation status and their altered gene expression in low- and high-grade non-muscle-invasive bladder cancer (NMIBC) subjects.

Materials and methods

This case-control study was conducted between 2020 and 2023, in which n = 115 tumor tissues (NMIBC n = 85) and (controls n = 30) were examined for FGFR3 and FGFR promoter methylation and expression using methylation-specific PCR (MSP) and real-time PCR. The multivariate regression analysis and Kaplan-Meier (KM) plots were used to establish the association of FGFR3 and TP53 with clinicopathological features and survival outcomes of NMIBC patients.

Results

High-grade NMIBC tumors showed substantial methylation patterns, with TP53 hypomethylated (p = 0.034) and FGFR3 hypermethylated (p = 0.046), as well as significant mRNA expression of Tp53 and FGFR3 (p = 0.001). The multivariate analysis shows FGFR3 and Tp53 were associated with recurrence-free survival with sensitivity (p = 0.045 (78%); 0.034 (70.7%)) and progression-free survival (p = 0.022(61.5%); 0.038 (69.2%)).

Conclusion

The findings of this investigation indicate that FGFR3 hypermethylation and TP53 hypomethylation are independent prognostic indicators that aid in the evaluation of disease outcomes in high-grade NMIBC tumors.

## Introduction

Bladder cancer (BC) is the second most frequently diagnosed cancer of the urinary system, with 5,73,000 new cases diagnosed globally in 2020 [1]. Initially, 70-80% of newly diagnosed BCs are non-muscle-invasive BC (NMIBC), with the histopathological staging of carcinoma in situ (CIS), pTa, and pT1 (2016 WHO Classification System) [2]. Furthermore, BC is classified into low-grade (LG) and high-grade (HG) non-invasive papillary urothelial carcinomas. The remaining 20-30% of BC are muscle-invasive BC (MIBC) with the histopathological stage of pT2-pT4 [3,4]. Primarily, transurethral resection of bladder tumors is used in combination with radiotherapy, chemotherapy, and immunotherapy such as mitomycin-C, or bacillus Calmette-Guerin (BCG) to manage BC [5]. However, BC shows a higher rate of recurrence (~70%) and progression (~40%) in NMIBC, which is associated with a poor therapeutic outcome [6].

Genetic and epigenetic changes influence the initiation and progression of cancer. Epigenetic changes are reversible modifications to DNA methylation, chromatin structure, and noncoding RNA profiles and are linked with the regulation of gene expression [6,7]. The epigenetic alterations linked to the recurrence and progression of BC. The hypomethylation and hypermethylation of DNA are linked to advanced tumor stages or increased incidence of BC [8,9]. Thus, understanding the regulatory system of gene expression is one of the most important steps to early diagnosis and possible therapeutic targets [10]. Additionally, epigenetic modification alters gene expression without changing DNA sequence, making it a more flexible and important therapeutic target for cancer treatment [9].

Fibroblast growth factor receptor 3 (FGFR3) and tumor protein 53 (TP53, most commonly known as P53), are crucial genes in human biology, playing distinct roles in cancer development, recurrence, and progression [11]. The FGFR3 gene is associated with cancers such as BC, multiple myeloma, and breast cancer [12]. Mutations, gene amplifications, and epigenetic regulation of FGFR3 lead to the activation of downstream pathways such as RAS-MAPK and PI3K-AKT and associated genes to promote uncontrolled cell growth leading to tumor formation [13]. However, p53 regulates the cell cycle, DNA repair, apoptosis, autophagy, and metabolism and maintains genome stability [14,15]. Primarily, P53 helps prevent cancer development by preventing cell proliferation with damaged DNA, either by promoting DNA repair or by inducing apoptosis. However, mutations and aberrant epigenetic regulation lead to uncontrolled cell division, which drives cancer progression and metastasis [16]. The aberrant FGFR3 and TP53 gene expression promote the initiation and progression of cancer. As a result, one possible target for cancer therapies could be changes in the FGFR3 and TP53 genes.

BC development as well as progression can result from abnormal DNA methylation, which can function as prognostic biomarkers, forecasting the course and likelihood of medication responses. It has been identified that the hypermethylated P16 (INK4a) gene has been significantly associated with disease recurrence, whereas hypermethylation of the RASSF1A gene has been linked with tumor progression [17]. Hypermethylation of death-associated protein kinase 1 (DAPK1) can be correlated with higher BC risk [18]. However, methylation of O6-methyl guanine DNA methyl transferase (MGMT), E-cadherin, and CDH1 genes cloud direct chemotherapy response, invasion, and aggressiveness of disease respectively. DNA methylation pattern can be used to predict BC recurrence, progression, and monitoring therapy response [19]. A study examining methylation patterns in BC found that hypermethylation of Janus kinase 3 (JAK3) and hypomethylation in EYA4, GATA6, and SOX1 was associated with LG intermediate-risk nonmuscle-invasive bladder cancer (LG NMIBC), while hypermethylation of CSPG2, HOXA11, HOXA9, HS3ST2, SOX1, and TWIST1 was linked to muscle-invasive bladder cancer (MIBC). A panel of hypermethylated genes, including APC, CSPG2, EPHA5, EYA4, HOXA9, IPF1, ISL1, JAK3, PITX2, SOX1, and TWIST1, was identified as a predictor of cancer-specific survival [20]. Further, hypermethylated SOX1, PITX2, or CSPG2 were associated with a higher risk of death from BC [20]. Research revealed a substantial variation in the methylation pattern of E2F1, ERBB2, HIC1, OPCML, SFN, SFRP1, SFRP2, SPARC, and TERT genes between control and BC samples. These findings highlight the need for population-specific studies to develop tailored biomarkers for BC [21].

The Tp53 and FGFR3 mutations are typically linked to a lower tumor recurrence [22]. Studies have shown that both LG and HG NMIBC use different molecular pathways to progress. LG NMIBC arise due to alteration in FGFR3 either by mutation or other mechanism, although HG NMIBC developed by loss-of-function mutations in the TP53 and RB1 genes [11,23,24]. Nevertheless, there is a lack of research on the relationship between the promoter methylation status of these genes and the prognosis of NMIBC. Therefore, we aimed to investigate the role of promoter methylation and its altered gene expression in the progression of NMIBC. The findings of this study have the potential for identification, management, and establishment of prognostic markers.

## Materials and methods

Demographic and clinicopathological characteristics

In this study, a total n = 115 individuals were enrolled. Tumor tissue (n = 85) from NMIBC patients and (n = 30); normal bladder mucosal tissues from BPH patients were obtained during transurethral resection of bladder tumor (TURBT) and biopsy from BPH patients. The study protocol was approved by the Institutional Ethics Committee (IEC) at King George’s Medical University, followed by a Declaration of Helsinki. The inclusion criteria are as follows: histopathological-confirmed NMIBC, followed by TNM and WHO 2004/2016 classification, stage (CIS, pTa-pT1), age (18-85 years), gender (male/female), smoking (yes/no), and willingness to participate. However, patients having diabetes, any inflammatory conditions (e.g., system lupus erythematosus, rheumatoid arthritis, sarcoidosis, Crohn's disease, and ulcerative colitis), and other types of cancer (including prostate cancer, kidney cancer, and upper tract urothelial carcinomas) were excluded. Patients were monitored up to 24 months from the date of enrollment (median: 14 months) to evaluate tumor recurrence and progression through cystoscopic examinations conducted every three months after initial TURBT for the first year and every six months after one year. If a tumor was detected during examinations, a biopsy and histological analysis were performed to determine tumor grade, T-stage, number, size, and whether it involved lymph nodes [25,26]. Tumor recurrence refers to the return of a disease after a period of successful treatment, whereas progression refers to the advancement of the disease, such as an increase in tumor grade (LG to HG), T-stage from (CIS or Ta to T1 or more than T2 stage), or lymph node involvement (N+) or distant metastasis (M1) seen on contrast-enhanced computed tomography (CECT)/histopathology report; then, the tumor was labeled as progression.

DNA extraction from NMIBC tumor and control tissues

Genomic DNA was extracted using a QIAGEN DNeasy Blood and Tissue kit (250; Cat no. # 69506), according to manufacturer instructions, and stored at −80°C until analysis. The extracted DNA samples were quantified with a Quawell-5000 UV-Vis Spectrophotometer (Quawell Technology Inc., San Jose), and integrity was assessed with agarose gel electrophoresis.

Methylation-specific polymerase chain reaction (MSP)

EZ DNA methylation gold kit (Cat no. # D5005; Zymo Research, Irvine, CA) was used for the bisulfite conversion of DNA. We used 500 ng of input DNA and 15 µL of elution buffer for the final elution and followed the rest of the procedure as instructed in the manual. The methylation status of FGFR3 and Tp53 genes was assessed with methylation-specific primers (Appendix Table 3) using bisulfite-converted DNA as a template. AmpliTaq Gold 360 Master Mix (439881; Applied Biosystems, Foster City, CA) was used for pathologic complete response (PCR) amplification with the following PCR cycling conditions: initial denaturation at 95℃ for five minutes, 35 cycles of (denaturation at 95℃ for 30 seconds, annealing at 59℃ for 30 seconds, extension at 72℃ for 20 seconds, and final extension at 72℃ for 10 mins. The reaction components included 1 µL of bisulfite-modified DNA (~33 ng), 5 µL of AmpliTaq gold master mix, 0.5 µL of FP (15 pmol), and 0.5 µL of RP (15 pmol) and make up the volume to 10 µL by adding 3 µL of nuclease-free water (NFW).

RNA extraction and cDNA synthesis

To determine the expression level of FGFR3 and Tp53 genes, total RNA was extracted from tissue samples using 1 mL/mg of TRIzolTM reagent (Cat No.15596026; Invitrogen, Carlsbad, CA). Quawell-5000 UV-Vis spectrophotometer (Quawell Technology Inc., San Jose, CA) was used to quantify RNA, samples having A260 nm/A280 nm absorption ratio > 1.8 were further analyzed. The first strand cDNA was synthesized from 1 microgram of the total RNA using a first-strand cDNA synthesis kit according to the manufacturer’s instructions (Promega Corporation, Madison, WI) and stored at -20˚C further analysis.

Real-time PCR

Real-time PCR was performed to analyze gene expression levels of FGFR3 and TP53 genes in both control and NMIBC tumors with Sybre green master mix, cDNA template, and RT-PCR primers (Appendix Table 4). The PCR cycling conditions included the following: initial denaturation at 95 ℃ for five minutes, followed by 40 cycles of amplification with denaturation at 95 ℃ for 15 seconds, annealing temperature for one minute, extension at 72 ℃ for one minute, and final extension at 72 ℃ for seven minutes (ABI Step-One Real-time PCR; Applied Biosystems). Each gene was tested in triplicate. Beta-actin was used as a reference gene to normalize the Ct value of a target gene. Further, ∆Ct values (∆Ct = Ct target - Ct reference) were calculated by comparing them with the mean Ct value of beta-actin. Furthermore, ∆∆Ct was calculated using ∆Ct of FGFR3 and TP53 in control tissue (∆∆Ct = ∆Ct tumor - ∆Ct reference). The average fold change of FGFR3 and TP53 gene expression in the NMIBC tumor was calculated using 2^ - ∆∆Ct [27].

Statistical analysis

The statistical values for the methylation index were defined by mean ± SD. Expression data were normalized, and one-way analysis of variance (ANOVA) was employed for analyzing the data and conducting intergroup gene expression comparisons. The survival curves were formed using the Graph Pad Prism 5 (GraphPad Software, San Diego, CA) software, and the log-rank test was used. Further univariate and multivariate analyses were performed with the Cox proportional hazards regression models with the Statistical Product and Service Solutions (SPSS, version 24; IBM SPSS Statistics for Windows, Armonk, NY). Pearson correlation was used to determine the correlation between DNA methylation and gene expression. P < 0.05 were considered as statistically significant.

## Results

Baseline characteristics of study participants

A total of n = 115 subjects were included, comprising n = 85 patients with NMIBC and 30 normal bladder mucosa tissues from individuals with benign prostatic hyperplasia (BPH) patients. Among these NMIBC patients, there were 73 (85.9%) males and 12 (14.1%) females. The mean age of NMIBC patients was 54.9 ± 11.01 years, while the mean age of the control group was 64.8 ± 11.2 years. The age distribution of NMIBC tumor patients was categorized into two groups: those who were younger than 60 years (n = 32, 37.6%) and those who were 60 years or older (n = 53, 62.4%). In contrast, the control group (n = 30; males) consisted of those younger than 60 years (n=4, 13.3%) and those who were 60 years or older (n = 26, 86.7%). Among the tumor patients, 64.7% had a history of smoking, while among the control patients, 56.6% had a history of smoking. On the other hand, 35.3% of the patients and 43.3% of the controls had never smoked. The detailed characteristics of individuals are listed in Table 1.

**Table 1:** Demographic and clinicopathological characteristics of NMIBC patients and control subjects (n = 115).

Variables	Controls (n = 30 (%))	NMIBC (n = 85 (%))
Age, years (mean)	64.8 ± 11.2	54.9 ± 11.01
≥ 60 years	26 (86.7)	53 (62.4)
< 60 years	4 (13.3)	32 (37.6)
Gender		
Male	30 (100)	73 (85.9)
Female	0	12 (14.1)
Smoking		
Yes	17 (56.6)	55 (64.7)
No	13 (43.3)	30 (35.3)
Number of tumors		
Single		32 (37.6)
Multiple		53 (62.4)
Size of tumors		
< 2.5 cm		22 (25.9)
≥ 2.5 cm		63 (74.1)
Tumor grade		
Low grade		33 (38.8)
High grade		52 (61.2)
Tumor stage		
pTa		31 (36.5)
pT1		47 (55.3)
CIS		7 (8.2)
Recurrence		
Yes		46 (54.1)
No		39 (45.9)
Progression		
Yes		13 (15.3)
No		72 (84.7)

DNA methylation of FGFR3 and TP53 in NMIBC

**Figure 1:**
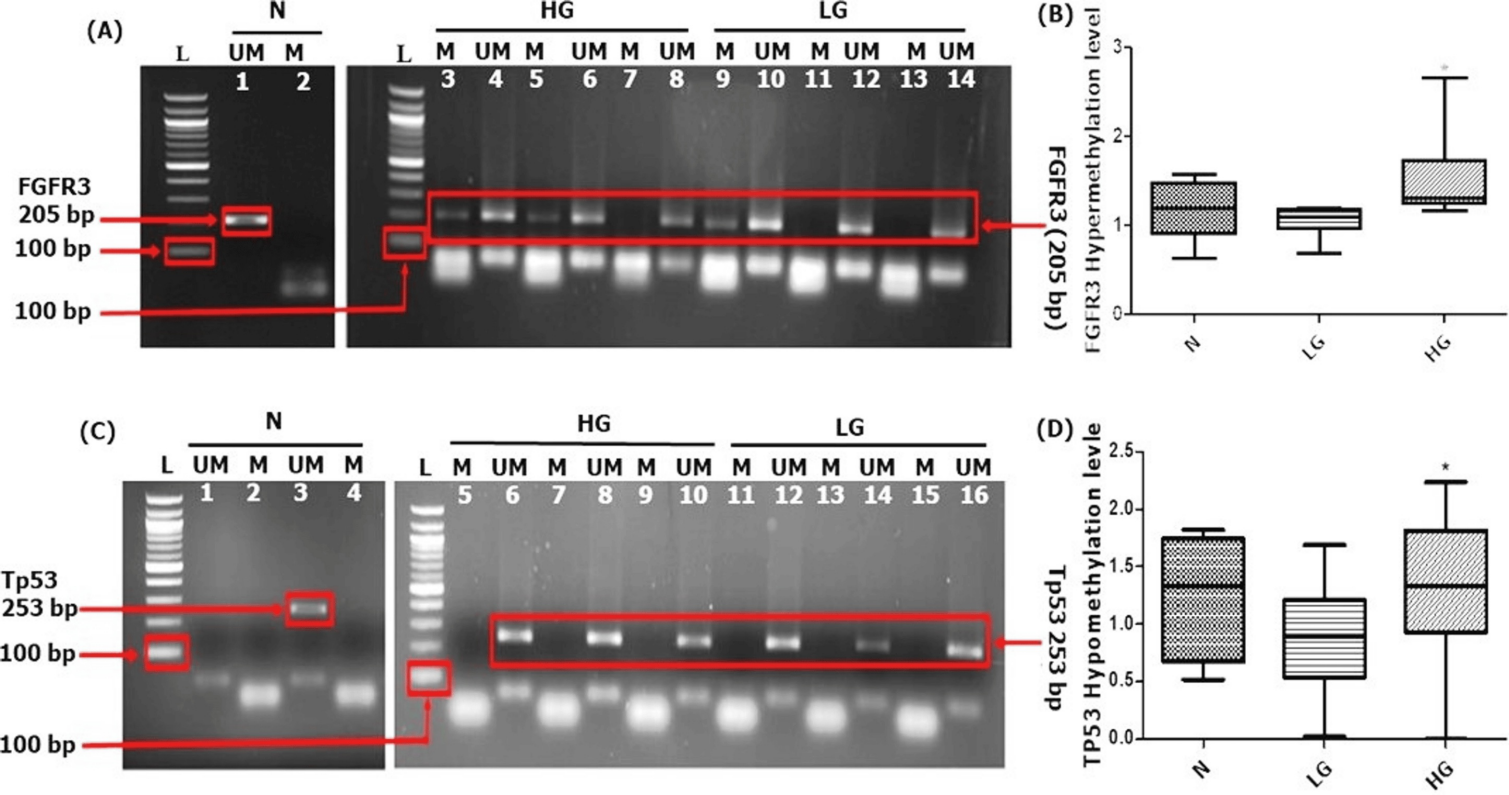
Methylation-specific PCR (MSP) in control and tumor NMIBC tissues. (a) FGFR3 (205 bp) lane 1 shows the unmethylated bands in the control group, whereas high-grade (HG) lanes 3 and 5 and low-grade (LG) lane 9 show the methylation. (b) The hypermethylation level of FGFR3 (p = 0.046) compared to the control. (c) TP53-methylation-specific bands in lane 3 show unmethylated in the control group. Additionally, lanes 6, 8, and 10 of high-grade tumors and lanes 12, 14, and 16 show unmethylation. (d) The hypomethylation level of TP53 genes between the control and HG tumors is statistically significant (p = 0.034). Here, P < 0.05 was considered as significant.

The promoter methylation of FGFR3 and Tp53 genes was examined using methylation-specific PCR in LG and HG NMIBC tumor and control tissue samples. The study's findings showed that both methylation patterns were present. Patients with HG NMIBC were shown to have hypermethylation of FGFR3 (1.841 ± 1.42) and hypomethylation of TP53 (2.40 ± 1.94). Furthermore, the association between clinicopathological characteristics and the distribution of methylation patterns were analyzed, and the results are shown in (Appendix Table 5). Moreover, we identified that HG NMIBC patients had substantial promoter hypermethylation of FGFR3 (p = 0.046), whereas HG NMIBC patients had hypomethylation of TP53 (p = 0.034) in comparison to LG patients and controls, respectively, as shown in Figure 1.

DNA methylation and the expression of the TP53 and FGFR3 genes in patients with LG and HG tumors

**Figure 2:**
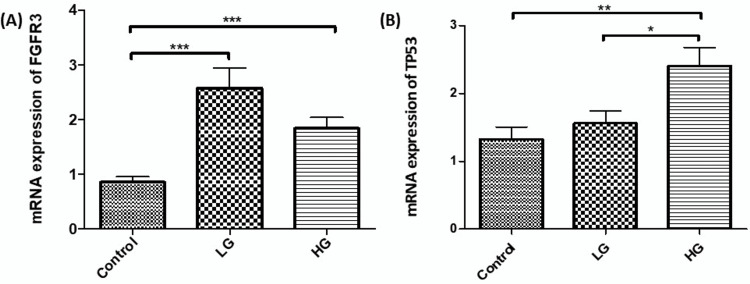
mRNA expression level of FGFR3 and TP53 genes in control and NMIBC tissues (grade). (A) FGFR3 gene and (B) TP53 gene expression analysis was performed, and significant differences (p-value) were identified using a non-parametric ANOVA test (p < 0.05, *), (p < 0.01, **), and (p < 0.001, ***).

To evaluate the DNA-methylation-induced gene silencing or activation, expression analysis for FGFR3 and Tp53 genes was performed in LG (n = 33), HG (n = 52), tumors, and control (n = 30) samples. The findings indicate a downregulation of FGFR3 and upregulation of TP53 in HG tumors, which shows a significant correlation as compared to control (P < 0.001), shown in Figure 2 (A: FGFR3 and B: TP53). Furthermore, Pearson correlation coefficient analysis revealed a strong association between the methylation and expression of FGFR3 (p < 0.05; r = -0.491) and TP53 (p < 0.05; r = 0.361) genes (Appendix Table 6).

Methylation status of FGFR3 and TP53 gene promoters as a predictor of survival

**Figure 3:**
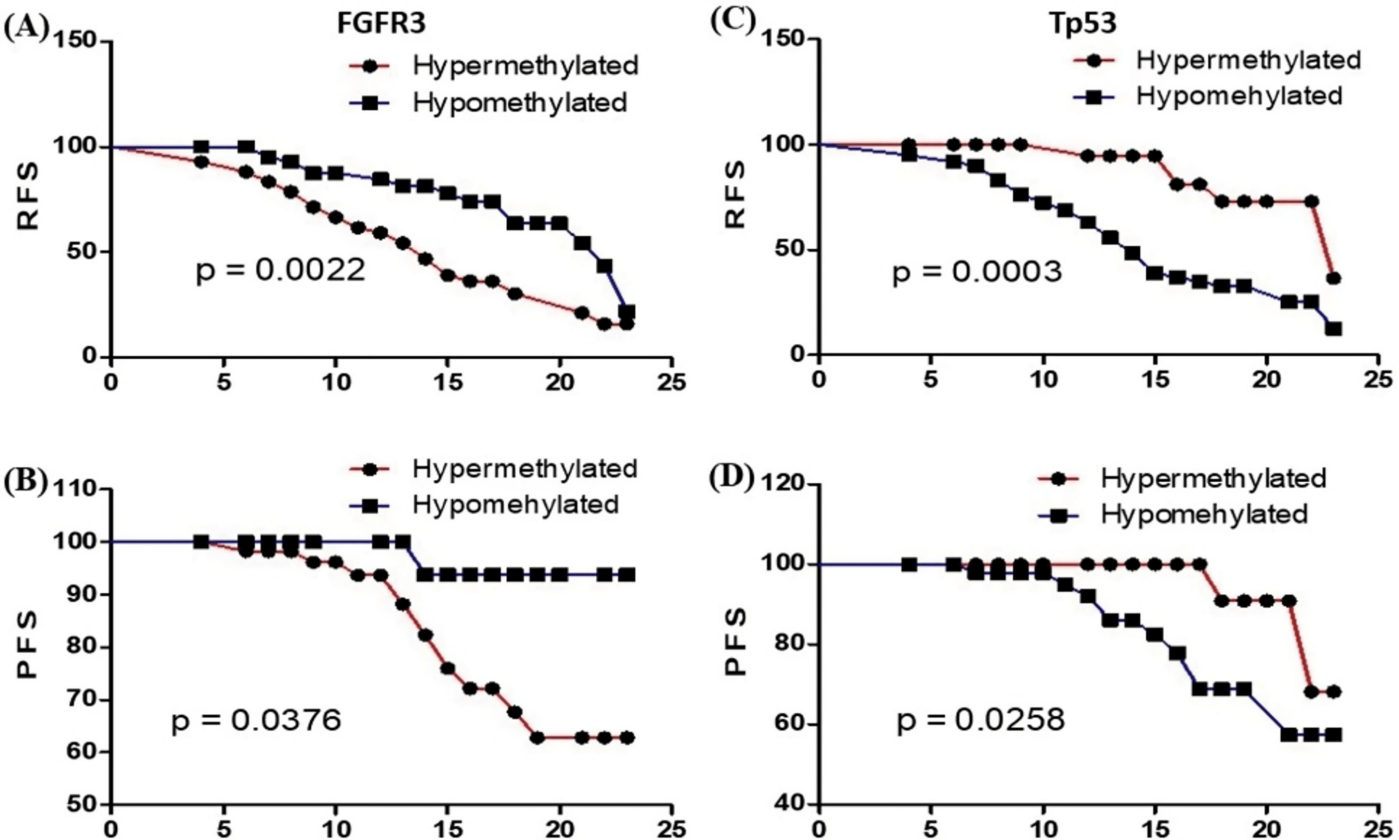
RFS and PFS in NMIBC patients were predicted using the Kaplan-Meier (KM) curve survival analysis. A & B): FGFR3 hypermethylation was linked to RFS (p = 0.002) and PFS (p = 0.037). However, (C & D) demonstrate PFS (p = 0.025) and RFS (p = 0.0003) for hypomethylated TP53.

The KM analysis was performed to identify the role of aberrant promoter methylation of FGFR3 and TP53 genes. This methylation index value was separated into two classes (hypermethylation and hypomethylation) with median cut-off points. Kaplan-Meier (KM) curve analysis identified significant differences between time to recurrence (FGFR3, p < 0.01; Tp53, p < 0.001) and progression (FGFR3, p < 0.05; TP53, p < 0.05) (Figures 3A-3D). Additionally, Cox proportional hazards regression analysis was conducted for both univariate and multivariate assessments of recurrence-free survival (RFS) and progression-free survival (PFS), as presented in Table 2. Results from the univariate and multivariate analysis revealed that hypermethylation of FGFR3 (p = 0.045; HR = 3.47) and hypomethylation of TP53 (p = 0.034; HR = 2.57) were significantly associated with RFS, while FGFR3 (p = 0.022; HR = 3.85) and TP53 (p = 0.038; HR = 1.78) were significantly associated with PFS. Further multivariate analysis showed that male gender (p = 0.008; HR = 9.97), HG (p = 0.043; HR = 2.97), stage pT1 (p = 0.031; HR = 0.08), and CIS (p = 0.025; HR = 0.37) can be independent prognostic markers for recurrence. In contrast, HG tumors (p = 0.042; HR = 3.99), stage pT1 (p = 0.043; HR = 2.48), and CIS (p = 0.038; HR = 2.07) are independent predictors of PFS in NMIBC patients. Furthermore, the importance of TP53 and FGFR3 methylation markers for predicting recurrence and progression was evaluated using receiver operating characteristic (ROC) curve analysis. The results indicated that FGFR3 has a sensitivity of 78.3% and specificity of 51.3% for RFS and a sensitivity of 61.5% and a specificity of 41.7% for PFS. Additionally, TP53 shows a sensitivity of 71.7% and specificity of 64.1% for RFS and a sensitivity of 69.2% and specificity of 31.9% for PFS. The ROC curve data are presented in Appendix (Table 7).

**Table 2:** Univariate and multivariate Cox regression analysis as a predictor of RFS and PFS. Univariate and multivariate Cox regression analyses were conducted to calculate the hazard ratio (HR) for each variable in predicting disease outcomes (RFS and PFS). The analysis considered methylation status (hypermethylation vs. hypomethylation) and various clinicopathological characteristics, including gender (male/female), age (≥ 60 years/< 60 years), smoking status (yes/no), tumor grade (high/low), tumor stage (pT1/CIS/pTa), tumor size (≥ 2.5 cm/< 2.5 cm), tumor number (multiple/single), and lymphovascular invasion (yes/no).

	Recurrence-free survival (RFS)		Progression-free survival (PFS)	
Variables	Univariate	Multivariate	Univariate	Multivariate
	HR (95% CI), P-value	HR (95% CI), P-value	HR (95% CI), P-value	HR (95% CI), P-value
Age	1.08 (0.600-0.1946), 0.797	1.33 (0.662-2.658), 0.426	1.33 (0.443-3.972), 0.61	1.59 (0.443-5.716), 0.477
Gender	5.48 (1.316-22.871), 0.019	9.97 (1.811-54.801), 0.008	3.37 (0.425-26.743), 0.25	4.24 (0.406-44.370), 0.228
Smoking	1.16 (0.620-2.193), 0.635	1.48 (0.706-3.078), 0.301	1.73 (0.470-6.345), 0.41	2.66 (0.492-14.401), 0.256
Tumor Grade	2.70 (1.301-5.607), 0.008	2.97 (1.037-8.465), 0.043	5.59 (1.831-17.083), 0.003	1.95 (0.249-15.202), 0.526
Tumor Stage	1.25 (1.028-1.525), 0.026	0.08 (0.009-0.802), 0.031	2.16 (1.106-4.215), 0.024	2.48 (1.031-5.957), 0.043
Tumor Stage	0.32 (0.153-0.664), 0.003	0.37 (0.155-0.883), 0.025	1.77 (1.048-2.992), 0.032	1.69 (1.015-2.082), 0.043
Size	1.23 (0.618-2.450), 0.556	0.64 (0.238-1.711), 0.372	4.19 (0.537-32.799), 0.172	0.64 (0.039-10.451), 0.755
Number	0.90 (0.476-1.724), 0.763	1.02 (0.486-2.148), 0.955	1.97 (0.425-9.162), 0.386	1.98 (0.305-12.848), 0.475
Invasion	1.13 (0.630-2.014), 0.689	0.72 (0.336-1.514), 0.379	0.85 (0.284-2.533), 0.768	0.37 (0.092-1.512), 0.167
FGFR3	2.46 (1.265-4.748), 0.008	3.47 (1.024-11.724), 0.045	1.76 (1.047-2.963), 0.033	0.41 (0.052-3.226), 0.036
Tp53	3.59 (1.373-9.381), 0.009	2.57 (1.051-6.284), 0.034	1.85 (1.145-2.975), 0.012	1.78 (1.035-3.054), 0.038

## Discussion

A limited number of studies were available to describe the clinical importance of aberrant DNA methylation in NMIBC [28]. Hence, we attempt to determine the clinical significance of the methylation and expression pattern of the FGFR3 and TP53 genes in the prognosis of NMIBC patients. The results show that HG tumors had significant hypermethylation and hypomethylation of the promoter FGFR3 (p < 0.05) and TP53 (p < 0.05), and the evaluation and co-relation of methylation-induced gene silencing or induction shows that the level of FGFR3 gene was significantly downregulated in HG tumor (p < 0.001) compared to LG tumors. A similar result was shown by Tomlinson et al. and Poyet et al., which shows that FGFR3 expression was upregulated in 80% of pTa whereas only 40-50% of tumors showed increased FGFR3 expression in pT1 tumors [29,30]. A study's findings show that regional hypermethylation of DNA in CpG C-type islands has accumulated in cancer-free kidney tissue, and changed DNA methylation may also detect malignant kidney lesions. DNA methylation in malignant kidney tissue may cause epigenetic alterations and genes, worsening tumors and affecting patient outcomes [31]. Additionally, other investigations demonstrated that a methylation test of NRN1, TERT, C228T, and FGFR3 for urothelial BC in urine sediment was developed. With single nucleotide polymorphism sites, the model was highly diagnostic. The model's AUC was high, such as fluorescence in situ hybridization (FISH) and conventional cytology. It could replace UBC detection [32].

In addition, the promoter region of the TP53 gene was found to be hypomethylation in both control and tumor tissues, and the degree of hypomethylation was significantly higher in HG tumors (p < 0.05) with co-related gene expression (p = 0.001). Meanwhile, a study shows that TP53 methylation was non-significant in BC [21]. However, aberrant methylation was seen at the promoter region of TP53 in leukemia patients and U937 cell lines (p = 0.018), glioblastoma, and chronic lymphocytic leukemia [33-35]. A similar result was shown by Borowczak et al. finding from this study demonstrated that TP53 expression was higher in cancer cases compared to controls (p = 0.021), HG vs LG (p = 0.08), and metastasis vs non-metastasis (p = 0.021) [36].

During the 24-month follow-up period, 54.12% (n = 46) patients showed tumor recurrence, whereas 15.3% (n = 15) had tumor progression. The increased methylation and downregulated or upregulated expression level of FGFR3 and TP53 genes were found to be associated with tumor recurrence (p < 0.01) and progression (p < 0.01) as compared to LG tumors. Furthermore, study results demonstrated that BC expressing decreased FGFR3 protein has a higher risk of cancer progression of HG tumors [30]. The study shows that P53 is overexpressed in BC cases in 22% (pTa), 46% (pT1), more than 71% (pT2-pT4), and 19% (G1), 38% (G30), and 74% (G3), respectively [37].

The Cox proportional hazard regression analysis based on the FGFR3 and TP53 promoter methylation status indicated that FGFR3 hypermethylation was significantly associated with RFS (HR = 3.47; p = 0.045) and PFS (HR =3.85; p = 0.022). A multivariate result shows that RASSF1A and DAPK methylation, tumor grade, and stage were significantly associated with disease progression (p = 0.04) [28].

Meanwhile, TP53 hypomethylation can be correlated with RFS (HR = 2.57; p = 0.034) and PFS (HR = 1.76; p = 0.038). The aberrant DNA methylation status of both genes may be considered as an independent prognostic marker for both RFS and PFS, respectively, in NMIBC tumors. Similarly, aberrant DNA methylation at FGFR3 and TP53 promoter in NMIBC shows that male gender (HR = 9.97; p = 0.008), HG (HR = 2.97; p = 0.043), and pT1 stage (HR = 0.08; p = 0.031) are an independent predictor of RFS, whereas HG tumor (HR = 3.99; p = 0.042) and pT1 stage (HR = 2.48; p = 0.043) are an important prognostic indicator for PFS. A Cox analysis shows that high FGFR3 expression was significantly associated with reduced RFS (HR = 3.78; p < 0.001) and improved OS (HR = 0.050; p = 0.043). KM results show that FGFR3 expression has reduced RFS in high FGFR3 expression (p = 0.001) [38]. An additional investigation revealed that the PlncRNA-1 gene's promoter was hypomethylated while having increased expression in BC. This suggests that the gene may have potential as a therapeutic target and a predictive biomarker for BC [39].

Limitation and strength 

Our study involved a relatively small sample size of 115 participants, which could potentially affect how applicable the findings are to a larger population. In addition, the study conducted at a single center may introduce selection bias and hinder the ability to establish causal relationships. The study's duration and follow-up period may not fully account for long-term changes in methylation patterns and gene expressions, which could impact the evaluation of survival outcomes. It focuses on FGFR3 hypermethylation and TP53 hypomethylation as potential prognostic markers for NMIBC. The study uses methylation-specific PCR and real-time PCR for accurate detection and quantification and employs multivariate regression analysis and KM plots for statistical evaluation. The findings have clinical relevance, predicting disease outcomes and guiding treatment decisions. The well-defined patient cohort ensures representative data collection.

## Conclusions

The methylation patterns of HG tumors (FGFR3 hypermethylation and TP53 hypomethylation) are associated with changes in mRNA expression. Furthermore, FGFR3 hypermethylation and TP53 hypomethylation serve as independent predictors of recurrence-free and progression-free survival in NMIBC. These findings suggest that these markers have the potential to be valuable prognostic indicators, providing insights into potential therapeutic strategies and personalized treatments for patients with HG NMIBC.

## Appendices

**Table 3:** List of forward and reverse primers used in the methylation-specific PCR study for each gene.

Gene		Forward/Reverse	Primer Sequence (5’-3’)
FGFR3	Methylated	F	5’-CGTTTTTTATTGGTTGCGGCGCGC-3’
		R	5’-CGCCGAAACCCGACTCGCTACGC-3’
	Unmethylated	F	5’-TGTTTTTTATTGGTTGTGGTGTGTG-3’
		R	5’-CACCAAAACCCAACTCACTACAC-3’
TP53	Methylated	F	5’-TTTTTGTTTTTTTCGGTAGGC-3’
		R	5’-CAAAAAAACGTATCACCGTC-3’
	Unmethylated	F	5’-TTTTTGTTTTTTTTGGTAGGTG-3’
		R	5’-AATCCAAAAAAACATATCACCATC-3’

**Table 4:** List of forward and reverse primers used in the real-time PCR expression investigation for each gene.

Gene	F & R	Primer Sequences	Fragment Size in (bp)	Tm (℃)
FGFR3	F	GGCCAGCAGGAGCAGTTGGT	182	58.0
	R	GAGTCCTCGTGGGAGGCATTC		
TP53	F	TCCCAGAATGCCAGAGGCTGCT	144	58.1
	R	AGACGGAAACCGTAGCTGCCCT		
ACTB	F	TACGAGCTGCCTGACGGCCA	209	59.0
	R	ATGCCAGGGTACATGGTGGTGC		

**Table 5:** Association between FGFR3 and TP53 methylation markers and clinicopathological characteristics of NMIBC patients. NMIBC: non-muscle-invasive bladder cancer

Variable	FGFR3		TP53	
	Hypermethylation Level (%)	p-value	Hypomethylation Level (%)	p-value
Gender		0.010		0.251
Female	1.1 ± 0.3		1.7 ± 0.8	
Male	1.5 ± 0.5		2.2 ± 1.0	
Age		0.065		0.360
< 60 years	1.2 ± 0.4		1.6 ± 0.6	
≥ 60 years	1.5 ± 0.3		1.8 ± 0.7	
Smoking		0.195		0.290
No	1.1 ± 0.4		1.1 ± 0.6	
Yes	1.4 ± 0.5		1.3 ± 0.5	
Tumor Number		0.065		0.088
Single	1.4 ± 0.3		1.8 ± 1.2	
Multiple	1.1 ± 0.4		1.5 ± 0.9	
Tumor Size		0.084		0.106
< 2.5 cm	1.3 ± 0.4		1.2 ± 0.8	
≥ 2.5 cm	1.9 ± 0.3		1.7 ± 1.1	
Lymphovascular Invasion		0.323		0.066
No	1.5 ± 0.7		1.7 ± 0.4	
Yes	1.6 ± 0.6		1.5 ± 0.3	
Tumor Grade		0.001		0.016
Low grade	1.3 ± 0.5		1.7 ± 0.7	
High grade	1.8 ± 0.3		1.4 ± 0.5	
Tumor Stage		0.002		0.003
Ta	1.7 ± 0.4		1.4 ± 0.3	
CIS	1.6 ± 0.4		1.3 ± 0.7	
T1	2.2 ± 0.8		1.7 ± 0.4	
Recurrence		0.018		0.022
No	2.2 ± 1.3		2.0 ± 1.0	
Yes	2.5 ± 0.9		2.2 ± 0.7	
Progression		0.036		0.024
No	1.35 ± 0.7		1.38 ± 1.1	
Yes	1.11 ± 0.4		1.32 ± 0.6	

**Table 6:** Pearson correlation analysis of methylation levels of GFR3 and TP53 genes associated with gene expression with each other in the tumor tissue of NMIBC patients. NMIBC: non-muscle-invasive bladder cancer

Genes	Correlation coefficient (r)	P-value
FGFR3	0.361	< 0.05
TP53	-0.491	< 0.05

**Table 7:** Receiver operating characteristic (ROC) curve to determine the diagnostic potential of FGFR3 and Tp53 genes. ROC curve of the methylation level of FGFR3 and TP53 genes in NMIBC tissue. NMIBC: non-muscle-invasive bladder cancer

Genes	Recurrence			Progression		
	AUC (95% CI)	Sensitivity (%)	Specificity (%)	AUC (95% CI)	Sensitivity (%)	Specificity (%)
FGFR3	0.687 (0.574-0.801)	78.3	51.3	0.709 (0.408-0.737)	61.5	41.7
TP53	0.612 (0.574-0.732)	71.7	64.1	0.572 (0.551-0.866)	69.2	31.9
